# Type I Interferons Are Associated with Subclinical Markers of Cardiovascular Disease in a Cohort of Systemic Lupus Erythematosus Patients

**DOI:** 10.1371/journal.pone.0037000

**Published:** 2012-05-14

**Authors:** Emily C. Somers, Wenpu Zhao, Emily E. Lewis, Lu Wang, Jeffrey J. Wing, Baskaran Sundaram, Ella A. Kazerooni, W. Joseph McCune, Mariana J. Kaplan

**Affiliations:** 1 Division of Rheumatology, Department of Internal Medicine, University of Michigan, Ann Arbor, Michigan, United States of America; 2 Department of Environmental Health Sciences, School of Public Health, University of Michigan, Ann Arbor, Michigan, United States of America; 3 Department of Biostatistics, School of Public Health, University of Michigan, Ann Arbor, Michigan, United States of America; 4 Department of Radiology, University of Michigan, Ann Arbor, Michigan, United States of America; Oklahoma Medical Research Foundation, United States of America

## Abstract

**Background:**

Systemic lupus erythematosus (SLE) patients have a striking increase in cardiovascular (CV) comorbidity not fully explained by the Framingham risk score. Recent evidence from *in vitro* studies suggests that type I interferons (IFN) could promote premature CV disease (CVD) in SLE. We assessed the association of type I IFN signatures with functional and anatomical evidence of vascular damage, and with biomarkers of CV risk in a cohort of lupus patients without overt CVD.

**Methodology/Principal Findings:**

Serum type I IFN activity (induction of five IFN-inducible genes; IFIGs) from 95 SLE patient and 38 controls was quantified by real-time PCR. Flow mediated dilatation (FMD) of the brachial artery and carotid intima media thickness (CIMT) were quantified by ultrasound, and coronary calcification by computed tomography. Serum vascular biomarkers were measured by ELISA. We evaluated the effect of type I IFNs on FMD, CIMT and coronary calcification by first applying principal components analysis to combine data from five IFIGs into summary components that could be simultaneously modeled. Three components were derived explaining 97.1% of the total IFIG variation. Multivariable linear regression was utilized to investigate the association between the three components and other covariates, with the outcomes of FMD and CIMT; zero-inflated Poisson regression was used for modeling of coronary calcification. After controlling for traditional CV risk factors, enhanced serum IFN activity was significantly associated with decreased endothelial function in SLE patients and controls (p<0.05 for component 3), increased CIMT among SLE patients (p<0.01 for components 1 and 2), and severity of coronary calcification among SLE patients (p<0.001 for component 3).

**Conclusions:**

Type I IFNs are independently associated with atherosclerosis development in lupus patients without history of overt CVD and after controlling for Framingham risk factors. This study further supports the hypothesis that type I IFNs promote premature vascular damage in SLE.

## Introduction

Systemic lupus erythematosus (SLE) is associated with strikingly high rates of premature cardiovascular disease (CVD) [1], [2], [3], [4]. A significant proportion of patients displays subclinical vascular disease [5], [6], [7] with premature and more severe coronary artery calcification and carotid intima media thickness (CIMT) and plaque [8], [9], [10]. Framingham risk factors are considered less important CVD predictors than active SLE [11]. While lupus immune dysregulation may play a dominant role in atherogenesis, the pathways implicated in accelerated CVD remain unclear.

As a potential mechanism explaining this enhanced risk, we reported that SLE patients develop a profound imbalance between endothelial cell (EC) damage and repair, manifested by increased circulating apoptotic ECs [5], decreased numbers and function of bone marrow derived endothelial progenitor cells (EPCs) and circulating angiogenic cells (CACs), and blunted synthesis of proangiogenic factors [12], [13]. Increased circulating apoptotic ECs in SLE correlates with endothelial dysfunction and tissue factor (TF) generation [5]. Similar EPC/CAC abnormalities have been identified as CVD risk factors in various diseases and in the general population [14], [15], [16].

Type I interferons (IFNs) appear to play major pathogenic roles in SLE [17] and their increased levels are reported in lupus blood and tissues [18], [19], [20]. We previously reported that type I IFNs induce abnormal EPC/CAC function in SLE. IFN-α promotes EPC/CAC apoptosis, skews CACs toward nonangiogenic phenotypes and represses proangiogenic molecule synthesis, including VEGF and IL-1β [13], [21]. Indeed, type I IFN pathway neutralization restores lupus EPC/CAC function [12]. Lupus EPC depletion and impaired arterial tone correlate with type I IFNs [22]. A proinflammatory subset of lupus neutrophils synthesizes increased type I IFNs and their depletion results in vasculogenesis improvements [23]. Further, IFN-α-dependent murine lupus models display endothelial dysfunction and aberrant vasculogenesis [24]. Recent evidence indicates that type I IFNs may also promote atherothrombosis by inducing a platelet proinflammatory phenotype [25] and foam cell formation *in vitro* [26]. These observations support a role for type I IFNs in lupus premature CVD.

The purpose of this study was to further characterize the role of type I IFNs in endothelial dysfunction and CV risk in lupus. To this end, we studied a cohort of SLE patients and controls without CVD history and with low burden of traditional CV risk factors, to examine the associations between serum type I IFN activity and various markers of vascular damage and atherosclerotic risk.

## Methods

### Objectives

The primary objective of this study was to examine the associations between type I IFNs and three measures of subclinical CVD – flow-mediated dilatation (FMD) of the brachial artery to assess endothelium-dependent vasorelaxation, carotid intima media thickness (CIMT), and coronary calcification, to assess subclinical atherosclerosis. Our hypothesis was that enhanced type I IFN activity would be associated with depressed FMD, and increased CIMT and coronary calcification, thereby promoting enhanced CV risk.

### Ethics

This study was approved by the University of Michigan’s Institutional Review Board. Study participants gave written informed consent.

### Participants

SLE patients meeting the revised American College of Rheumatology (ACR) classification criteria for SLE [27] were recruited from the Michigan Lupus Cohort [28], and were eligible for inclusion if they were <55 years of age at baseline and had no previous history of overt CV events. Patients were excluded if they were smokers (currently or within the previous 6 months), had diabetes, were pregnant, had a current infection, or were taking more than one antihypertensive in addition to a diuretic. Controls fulfilling the same eligibility criteria were recruited from the University of Michigan Women’s Health Registry [29], and were frequency matched by age and sex to the SLE patients.

### Description of Procedures Undertaken

#### SLE-specific measures

SLE activity was prospectively measured with the SLE Disease Activity Index (SLEDAI) and accumulated damage by the Systemic Lupus International Collaborating Clinics/ACR (SLICC/ACR) Damage Index [30], [31].

#### Vascular function assessment

FMD was quantified by ultrasound as previously described [5], [28]. Brachial artery diameter (BAD) baseline measurements were obtained. A blood pressure (BP) cuff was inflated to 50 mmHg above participant’s systolic BP over proximal portion of right forearm for 4 minutes. FMD was determined 1 minute after cuff release. Image acquisition was gated to EKG’s R wave. The endpoint was the percentage change in mean BAD in response to reactive hyperemia (FMD).

#### Carotid ultrasound

CIMT was assessed by B-mode ultrasonography. One centimeter segments of bilateral common carotid arteries, carotid bulbs, and distal internal carotid arteries were scanned. Images were obtained of the near or far wall of each arterial segment per standardized procedures. Intima-medial complex thickness was calculated as the distance from leading edge of first echogenic line to second echogenic line as described [32]. Final “mean CIMT” represents the average of 12 measurements made at end diastole, corresponding to distances between lumen-intima and media-adventitia interfaces on both sides, at the far and near arterial wall at 3 points within a 1.5-cm segment immediately caudal to carotid bulb. “Maximal CIMT” represents the maximal value of the 12 measurements. Wall thickness was measured outside atherosclerotic plaques (if present).

#### Coronary calcification

Coronary calcium CT examinations were acquired on a GE LightSpeed™ QXi, LightSpeed 16, LightSpeed 16 Pro or VCT multidetector scanners of 4, 16 and 64 detector rows respectively. Analysis was performed using GE SmartScore PRO™ Coronary Artery Calcification Scoring software on a GE Advantage Windows workstation version 3.1–4.1 (GE Medical Systems, Milwaukee, WI). Prior to scanning, a 3-lead EKG was applied to patients for gating and the signal optimized. Anteroposterior and lateral scout images localized the heart. Images were obtained at 2.5 mm collimation from cardiac apex to base in cine mode with gantry rotation time of 0.35–0.6 seconds during a single breath-hold using a 25 cm field of view, 120 kVp and mA between 250 and 550 depending on patient size, at end diastole. From overlapping images series, the least motion-affected image within each scan group was selected automatically for calcification scoring. SmartScore computed the calcium score using standardized scoring techniques [33]. Generated scores were the AJ-130 total calcium and the volume scores.

#### Circulating markers of vascular damage

Soluble monocyte chemoattractant protein-1 (MCP-1), intercellular adhesion molecule-I (ICAM-1), vascular cellular adhesion molecule-1 (VCAM-1), tissue factor (TF), vascular endothelial growth factor (VEGF) (R&D systems, Minneapolis, MN); von Willebrand factor, plasminogen activator inhibitor-1 (PAI-1) and tissue plasminogen activator (TPA) (American Diagnostica, Stamford, CT) were quantified by ELISA. High sensitivity CRP (hsCRP) was quantified by ultra-sensitive rate immunonephelemetric method (Dade-Behring, Marburg, Germany).

#### Serum type I IFN activity

Control and lupus serum was assayed for the capacity to induce IFN-inducible genes (IFIGs) on epithelial cell lines using a validated bioassay [34], previously used by us [12]. Individuals with recent infections were excluded. CRP and white cell counts were used to exclude subclinical infection. Induction of IFIGs (*IFI44, IFI44L, MX-1, IFIT1 and PRKR*) on HeLa cells was quantified by real-time PCR [12]. Samples were normalized to media alone after normalization to house-keeping gene *HPRT-1* and results reported as fold induction/media.

#### Other measures

CVD family history was considered positive if a first-degree relative had a CV event at age <55 or 65 years for male and female relatives, respectively. Lipids, CBC, urine analysis, serum chemistry and homocysteine were quantified at University of Michigan Central Laboratories. C3 and C4 were measured using immunoturbidimetric methodology (Roche Diagnostics, Indianapolis, IN); anti-dsDNA by Farr radioimmunoassay (Diagnostic Products Corporation, Los Angeles, CA); IgG and IgM anticardiolipin and IgG, IgM and IgA anti-β-2 glycoprotein-I Abs by ELISA and lupus anticoagulant (LA) by dilute Russell viper venom test (American Diagnostica). SLE patients underwent determinations of antiphospholipid antibodies and LA at least twice; among patients with positive values at baseline, a second determination at least 6 weeks apart was performed.

### Statistical Methods

Summary statistics were computed for continuous measures as mean ± standard deviation or median (interquartile range, IQR) if the distribution was skewed, and for categorical variables as frequency and proportion (%). Variables were assessed for normality and log-transformed if needed. Pearson or Spearman’s rank correlations were used to examine associations between continuous variables. Correlation results were adjusted for multiple comparisons using Holm’s method [35]. Two sample t tests or Kolmogorov-Smirnov testing were used for comparison of continuous variables between groups, and the Chi-square test for comparison of proportions.

#### IFIG data

We first standardized each of the five IFIGs using their respective means and variances, so that the measurements had the same scale. Our primary analysis utilized principal component analysis (PCA) to combine the five standardized IFIGs together in a few linear combinations, in order to (a) avoid multi-collinearity between the IFIGs, (b) simultaneously account for the majority of the total system variability in the five IFIGs, and (c) reduce the number of parameters to estimate in the model. Since the generated principal components are orthogonal to one another, they can be modeled together without multi-collinearity issues that would be encountered by simultaneous modeling the five raw IFIG variables.

Univariate and multivariable linear regression were used to examine the association of principal components generated from the PCA and other predictor variables on the outcomes of FMD and CIMT; zero-inflated Poisson (ZIP) regression was utilized for the outcome of coronary calcification score due to the large proportion of zero values for this outcome variable. Main effects, as well as their interactions with SLE/control status, were included in the multivariable models. Variables that we adjusted for in the multivariable modeling included Framingham score, hsCRP, PAI/TPA, tissue factor, SLE disease duration, and current medication use (prednisone, antimalarials, statins, immunosuppressives). Because the PCA components of IFIGs were of our main interest, we made the *a priori* decision to retain them in the models regardless of significance. For non-PCA covariates (eg, traditional CV risk factors and medications), we removed variables that were strongly correlated to one another to prevent collinearity issues. PCA components’ interactions with SLE case/control status found to be non-significant at the 0.10 level were removed from the models during the model building process. *P* values <0.05 in the final models were considered statistically significant. All statistical analyses were performed using SAS 9.2 and R software.

## Results

### Participant Characteristics

A total of 95 SLE patients and 38 controls were included in this study. Patients and controls were similar in terms of race/ethnicity, traditional CV risk factors (including Framingham score), and CVD family history. A small proportion of SLE patients were positive for antiphospholipid antibodies (anticardiolipin, lupus anticoagulant, and/or beta2-glycoprotein I) **(** **Table 1** **)**. Concomitant therapies for SLE patients included: prednisone in 58 patients (61.1%, mean dose 9.2±8.2 mg/day); antimalarials in 59 (62.1%); methotrexate in 8 (8.4%); azathioprine in 10 (10.5%); mycophenolate mofetil in 21 (22.1%); cyclophosphamide in 4 (4.2%). Forty-four patients (46.3%) were on NSAIDS and/or aspirin; and 8 (8.4%) were on a statin. When compared to controls, SLE patients had decreased brachial artery FMD (4.0±4.7 patients vs 5.7±4.1 controls; p = 0.05), indicating decreased endothelial function. There were no significant differences in CIMT or coronary calcification at study enrollment between SLE and controls (**Table 1** **)**.

**Table 1:** Baseline characteristics for SLE patients and controls.

Variable	SLE (n = 95) Mean±SD orMedian (IQR) or No. (%)	CONTROLS (n = 38) Mean±SDor Median (IQR) or No. (%)	P value
Females	95 (97.9%)	38 (100%)	NS
Age (years)	37.6±9.1	39.3±10.2	NS
Race			NS
White	80 (84.2%)	34 (89.5%)	
Black	12 (12.6%)	1 (2.6%)	
Other	3 (3.2%)	3 (7.9%)	
Framingham score	2.8±6.1	3.7±6.5	NS
BMI (kg/m^2^)	25.0±5.3	27.3±6.1	NS
Systolic BP (mmHG)	119.6±15.5	124.3±13.9	NS
Diastolic BP (mmHG)	73.6±10.6	72.7±13.9	NS
Total cholesterol (mg/dl)	188.1±47.4	198.6±47.1	NS
Triglycerides (mg/dl)	89 (62, 134)	82 (66, 114)	NS
High density lipoproteins (mg/dl)	60.5 (50, 74)	58 (46, 66)	NS
Low density lipoproteins (mg/dl)	98 (80, 125)	110 (91, 130)	NS
Family history of CVD	23 (24.5%)	12 (31.6%)	NS
Homocysteine (umol/L)	7 (6, 10)	6 (5, 7.5)	NS
hsCRP	1.3 (0.7, 3.6)	2.4 (0.9, 5.8)	0.05
TPA/PAI-1	2.4 (1.2, 4.2)	3.0 (1.6, 4.7)	NS
Tissue Factor	127.7 (90.5, 168.3)	112.7 (72.1, 179.8)	NS
***SLE-specific measures***			
SLEDAI score	4 (0, 6)	–	–
Damage index	0 (0, 1)	–	–
PGA (0–3 visual analog scale)	0.1 (0, 0.5)	–	–
aCL - IgG †	4 (4.2%)	–	–
aCL - IgM †	2 (2.1%)	–	–
Lupus anticoagulant †	11 (11.6%)	–	–
anti-β2GPI - IgG †	1 (1.1%)	–	–
anti-β2GPI - IgM †	4 (4.2%)	–	–
anti-β2GPI - IgA †	7 (7.4%)	–	–
***CVD surrogates***			
FMD (% change)	4.0±4.7	5.7±4.1	0.05
Mean CIMT (mm)	0.58±0.11	0.57±0.12	NS
Maximal CIMT (mm)	0.69 (0.59, 0.82)	0.65 (0.60, 0.77)	NS
Coronary calcification scor			
Proportion positive (score >0)	22/95 (23.2%)	8/38 (21.1%)	NS
Score among positives	2.5 (1, 73)	10.5 (2, 28)	

SD: standard deviation; IQR: interquartile range; CIMT: carotid intima media thickness; aCL: anticardiolipin antibodies; anti-β2GPI: beta2-glycoprotein I; PGA: physician global assessment, BP: blood pressure; BMI: body mass index; NS: not significant; FMD: flow mediated dilatation.

† Antiphospholipid antibodies considered positive if positive on ≥2 occasions.

### Association of Serum IFN Activity with Surrogate Markers CVD

In our study population, levels of the five individual IFIGs were not statistically different between SLE patients and controls, although *MX-1* and *IFI44* levels were higher among lupus patients [median (IQR) expression levels: 4.4 (0.4, 48.5) in SLE versus 0.9 (0.3, 17.0) in controls; 5.3 (0.9, 55.4) in SLE versus 4.0 (0.7, 49.0) in controls, respectively]. Consistent with previous publications [19], [36], [37], there was considerable variability and a wide range of IFIG values.

**Figure 1:**
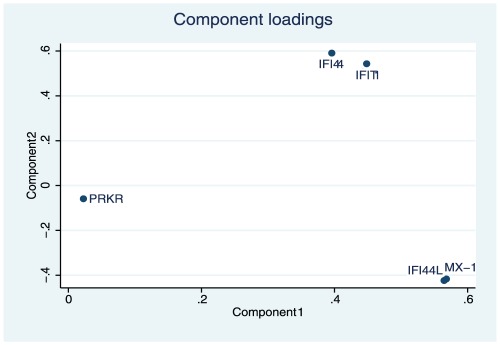
Plot of the first two principal component loading vectors. Each point represents one of the five IFIGs**.** Proximity of points illustrates the groupings of the IFIGs with respect to the principal components. The x-axis is the principal component factor loading for the first principal component (representing the coefficient for each IFIG in the linear combination as the principal component); the y-axis is the principal component factor loading for the second principal component. The first component can be interpreted approximately as the sum of information from *MX-1*, *IFI44L*, *IFIT1*, and *IFI44*. The second component can be interpreted approximately as the difference between the sum of (*IFIT1* and *IFI44*) and the sum of (*MX-1* and *IFI44L*).

From the PCA on IFIGs, we found that the first three principal components explain 97.1% of the variance information in the original five IFIGs. Therefore, these three principal components were used in the multivariable modeling. **Figure 1** shows the principal component factor loadings for the first two principal components, which visually depicts IFIG groupings within the first two principal components. The x-axis of Figure 1 is the principal component factor loading (which is the coefficient for each IFIG in the linear combination as the principal component) for the first principal component. As shown, the x-axis of *MX-1*, *IFI44L*, *IFIT1*, and *IFI44* are very close to each other, while the one for *PRKR* is very close to 0. As such, the first principal component is a linear combination of the five IFIGs with the coefficients for the first four being about the same and *PRKR*’s coefficient being about 0. Therefore, the first component can be interpreted as approximately the sum of information from *MX-1*, *IFI44L*, *IFIT1*, and *IFI44* (≈*IFIT1+IFI44+MX-1+IFI44L*). Similarly, the y-axis in Figure 1 is the principal component factor loadings for the second principal component, where we see the loading for *PRKR* is still close to 0, while those of *MX-1, IFI44L, IFIT1*, and *IFI44* have about the same absolute values but the first two are negative and the latter two are positive. Therefore, the second component can be interpreted as approximately the difference between the sum of *IFIT1* and *IFI44* and the sum of *MX-1* and *IFI44L* [≈(*IFIT1+IFI44*)*−*(*MX-1+IFI44L*)]. Further (not shown in Figure 1), the third component has factor loadings for IFIGs all very close to 0 except *PRKR*, and thus can be interpreted approximately as *PRKR* by itself (≈*PRKR*). Data from separate multivariable models for each CVD surrogate outcome are summarized below; the regression coefficients and 95% CIs for the PCA approach are presented in **Table 2** **.** The analyses included both cases and controls for CIMT and FMD, while the multivariable modeling for coronary calcification was restricted to SLE group, since there were too few controls with positive calcification scores to enable meaningful inclusion for that outcome.

**Table 2:** Results from multivariable regression models investigating the association between serum type I IFN activity and measures of subclinical CVD.

	SLE Cases & Controls			SLE Cases Only
	FMD†	Mean CIMT†	Maximal CIMT (log)†	Calcification Score‡
	β coeff (95% CI)	β coeff (95% CI)	β coeff (95% CI)	β coeff (95% CI)
IFIG Comp 1^a^	0.281 (−0.269, 0.831)	0.019 (0.006, 0.031)**	0.035 (0.002, 0.067)*	−0.868 (−2.129, 0.393)
IFIG Comp 2^b^	0.515 (−0.224, 1.254)	0.033 (0.008, 0.058)*	0.072 (0.010, 0.135)*	−0.868 (−0.052, 1.788)
IFIG Comp 3^c^	−12.423 (−23.888, −0.958)*	−0.353 (−0.880, 0.174)	−0.711 (−2.029, 0.607)	61.301 (57.222, 65.380)***
Control (vs. SLE)	2.489 (−0.296, 5.274)	0.104 (0.009, 0.199)*	0.278 (0.040, 0.515)*	–
Interactions				
Comp 1 × Control	–	–	–	–
Comp 2 × Control	–	−0.206 (−0.317, −0.094)***	−0.432 (−0.710, −0.153)**	–
Comp 3 × Control	–	0.580 (−0.032, 1.192)	1.602 (0.071, 3.132)*	–

Principal components analysis was first utilized to combine data from five IFN-inducible genes (IFIGs) into three principal components (explaining 97.1% of the total IFIG variation) that could be simultaneously included as independent variables in the multivariable models. Data are presented for separate multivariable models (separate columns), according to outcome.

a Principal component 1 (≈*IFIT1*+*IFI44*+*MX*-*1*+*IFI44L*);

b Principal component 2 [≈(*IFIT1*+*IFI44*)−(*MX*-*1*+*IFI44L*)];

c Principal component 3 (≈*PRKR)*. Note: IFIGs scaled by factor of 100.

* p<0.05;

** p<0.01;

*** p<0.001.

† controlled for Framingham score, disease duration, and current medication use of prednisone, antimalarial, and statin; additional modeling controlling for hsCRP, PAI1/TPA, tissue factor, yielded similar results (data not shown).

‡ SLE only models adjusted for Framingham score, current medication use of prednisone, antimalarial, and statin, plus SLEDAI and PAI-1/TPA; additional modeling including immunosuppressive use (cyclophosphamide, azathioprine, mycophenolate mofetil) yielded similar results (data not shown).

#### FMD

The association between FMD and the first three components derived from the IFIG PCA were examined. Based on the multivariable modeling, a 100 unit increase in the expression level of component 3 (≈*PRKR*) was associated with an average decrease in FMD (expressed as % change of the brachial artery diameter in response to reactive hyperemia) of 12.4 units (p<0.05), when controlling for Framingham score, disease duration, and baseline medication use of prednisone, antimalarial, and statins (see **Table 2**). The Framingham score was associated with depressed FMD (β coefficient −0.14; p = 0.04), while the remainder of the variables in the model were not associated with FMD. Additional modeling, controlling for hsCRP, PAI1/TPA, and tissue factor, yielded similar results and results from models excluding medication use were also similar (data not shown). These results indicate that enhanced serum IFN activity is associated with decreased endothelial function in SLE patients and controls.

#### CIMT

When mean CIMT was modeled as the outcome, significant associations with the IFIG components 1 (≈*IFIT1+IFI44+MX*-*1+IFI44L*) and 2 [≈(*IFIT1+IFI44*)*−*(*MX*-*1+IFI44L*)] were observed. In the multivariable model (see **Table 2**), a 100 unit increase in the expression level of component 1 was associated with a statistically significant increase of the mean CIMT for SLE cases by 0.02 mm (p<0.01) when controlling for all other variables (Framingham score, disease duration, and baseline medication use of prednisone, antimalarial, and statins). This association is not statistically different for the controls. A 100 unit increase in the expression level of component 2 was associated with a statistically significant increase of the mean CIMT for SLE cases by 0.03 mm (p<0.01). In contrast, for controls, a statistically significant negative association was seen, such that for every 100 unit increase in the expression level of component 2, there was a corresponding decrease in mean CIMT of 0.17 mm (*i.e.*, −0.17 = 0.03−0.20; p<0.001). Among the other variables in the model, the Framingham score was positively associated with mean CIMT (β coefficient 0.01; p<0.001) and antimalarial use was positively associated at borderline statistical significance (β coefficient 0.05; p = 0.05). Additional multivariable models, controlling for hsCRP, PAI1/TPA, and tissue factor, yielded similar results and results from models excluding medication use were also similar (data not shown).

As shown in **Table 2**, results for the outcome of maximal CIMT were similar to those for mean CIMT. When controlling for Framingham score, disease duration, and baseline medication use of prednisone, antimalarial, and statins, components 1 and 2 were positively associated with maximal CIMT for the SLE cases, whereby a 100 unit increase in expression of each of these components was associated with statistically significant increases in the log maximal CIMT of 0.04 log(mm) (p<0.05) and 0.07 log(mm) (p<0.05), respectively. For controls, component 2 was negatively associated with the maximal CIMT, where a 100 unit increase in expression for component 2 significantly decreased the maximum log CIMT by 0.36 units (*i.e.*, *−*0.36 = *−*.43+0.07; p<0.01). Conversely for controls, a statistically significant association between component 3 (≈*PRKR*) and maximal CIMT was observed for controls, where a 100 unit increase in expression for component 3 was associated with an increase in the log maximal CIMT of 0.89 log(mm) (p<0.05). These results indicate that enhanced serum IFN activity, as represented by components 1 and 2, is associated with increased CIMT in SLE, but that the association between component 2 and CIMT was reversed in the non-SLE controls. However, the positive association between *PRKR* and maximal CIMT observed in controls further implicates an independent role for *PRKR* since, as shown above, it was also associated with impaired brachial reactivity. Thus, the FMD and CIMT results together suggest a distinctive nature of *PRKR* with regards to association with CV measurements, in contrast to the other four IFIGs examined in this study (*IFIT1*, *IFI44*, *MX-1*, and *IFI44L*).

#### Coronary calcification

As noted above, modeling of coronary calcification was restricted to SLE patients since only 8 controls had non-zero values for the coronary calcification score outcome. 22 of the 95 SLE patients had non-zero calcification scores. The zero-inflated Poisson (ZIP) regression modeling includes two parts – the first of which is a logistic model where the calcification score outcome is handled as a binary variable (positive or negative) in the full SLE group of 95 patients, whereas the second part is a Poisson model examining the calcification score level as a continuous measure among the subset of 22 patients with a positive score. When examining the risk of having a positive coronary calcification score (as a binary variable), we found the Framingham score (OR 1.3, 95% CI 1.1, 1.5) and PAI-1/TPA (OR 1.3, 95% CI 1.0, 1.6) were associated with a positive coronary calcification score in the multivariable model. In the second part of the ZIP modeling, which was restricted to the 22 SLE patients with positive coronary calcification scores, a statistically significant association between principal component 3 (≈*PRKR*) and level of coronary calcification was detected when controlling for all other variables in the model, whereby each 1 unit increase in expression of principal component 3 was associated with a 1.8-fold increase in expected coronary calcification score [*i.e.*, exp(61.301/100) = 1.8; p<0.001; the exponential of the coefficient in the Poisson regression model represents the ratio of the expected coronary calcification scores associated with increase of the corresponding variable]. However, in this restricted portion of the model, the Framingham score, PAI-1/TPA, SLEDAI, and prednisone were all negatively associated with the level of coronary calcification (p<0.01), while statin use was positively associated (p<0.01). Additional modeling including immunosuppressive use (cyclophosphamide, azathioprine, mycophenolate mofetil) yielded similar results (data not shown). These results indicate that traditional risk factors, but not IFN serum activity, are associated with risk of having a positive coronary calcification score. However, among those SLE patients with a positive coronary calcification score, the traditional risk factors are negatively associated and IFN serum activity is positively associated with the severity of coronary calcification. These results may indicate the possibility of a differential effect of various risk factors for the initial development and progression of calcification in patients with SLE.

## Discussion

In this study we demonstrate that, in a cohort of SLE patients with on average low disease activity and without pre-existing CVD, non-traditional risk factors are independently associated with preclinical vascular damage. After controlling for known CV risk factors, serum type I IFN activity is independently associated with decreased FMD and with increased CIMT and coronary calcification severity in SLE. These observations further support the hypothesis that type I IFNs may play a pathogenic role in premature CVD in SLE.

This study builds upon previous suggestions of a role for type I IFNs in CVD [12], [22], by refining the approach towards examining IFIGs as a system. As recently noted by Reynier *et al*., the common approach of calculating a binary “IFN score” (high versus low) based on average gene expression levels ignores co-regulation of such IFN-related genes, which in turn can lead to misclassification [38]. The high degree of collinearity between IFIGs’ induction also precludes simultaneous modeling of the individual genes. PCA enabled the transformation of the individual correlated IFIGs into a smaller number of uncorrelated variables, the principal components, which account for as much of the data variability as possible. We thus derived composite measures of the five individual IFIGs without collinearity or arbitrary, *post hoc* decisions regarding creation of a composite IFIG measure or removal of outliers. The resulting variables representing IFIGs can be modeled simultaneously, while retaining as much information as possible.

Results from our systems-based approach indicate that levels of *PRKR* induction were independently associated with impaired FMD in both cases and controls, increased maximal CIMT in controls, and increased coronary calcification among SLE patients (this last outcome could not be studied among controls due to few having positive calcification scores). In contrast, the effects of the other four IFIGs–*MX-1*, *IFI44L, IFIT1*, and *IFI44*– appear to impact the vasculature in an orchestrated fashion. In particular, induction of these IFIGs was associated with increased CIMT in SLE patients, but decreased CIMT among controls. The basis for this observed interaction between these IFIGs and case/control status is unclear, and warrants further investigation. However, the presence of such interactive effects supports the concept that the excess CVD risk in SLE may be partially attributable to SLE-specific factors. This finding of differential effects associated with different IFIGs could indicate that various vascular territories respond differently to immunologic insults; this is supported by studies in the general population [39]. The observed differential effects also underscore the need to follow an approach such as PCA, as the use of a single binary summary score integrating data from the various IFIGs would be an oversimplification and would dilute results. Indeed, as an exploratory measure we calculated a high versus low IFN score (a “high” score representing individuals with at least 2 IFIGs above the 95^th^ percentile among controls for each IFIG). We found that 15.8% of SLE patients and 7.9% of controls were classified as “high producers” according to this classification. While we replicated the finding that CIMT was associated with high IFN production in SLE patients, we did not detect associations with endothelial dysfunction or coronary calcification when using this binary classification.

While recent evidence suggests that type I IFNs could play a role in “idiopathic” atherosclerosis in the general population, our results underscore the utility of following a systems-based approach to more specifically characterize the components of the type I IFN pathway that may be involved in vascular damage. Available data indicate that IFN-α levels are increased in atherosclerotic plaque [40], [41]. LDL-receptor deficient mice exposed to recombinant IFN-α develop worsening hyperlipidemia and atherosclerosis, while recent evidence points at a deleterious role of another type I IFN, IFN-β, in murine atherosclerosis [42], [43]. IFN-α may play an immunostimulatory role in the atherosclerotic plaque by enhancing vascular damage by T and NK cells [40]. There is a potential association between type I IFNs and platelet activation and foam cell formation [26], [44], which could enhance thrombogenicity and plaque deposition and predispose to acute coronary syndromes.

Whether activation of the specific IFIGs directly induces vascular effects, or whether type I IFN activation may impact the vasculature via other pathways, remains to be identified. However, the associations we detected are relevant to both functional (FMD) and anatomic (CIMT, coronary calcification) measures of subclinical CVD, and various stages in the natural history of CVD. This raises the possibility of various targets and windows for intervention.

Another contribution of this study is that controls were selected from the general population, according to same eligibility criteria as SLE patients, rather than restricting them to “healthy” individuals. SLE patients were included irrespective of disease severity, rather than restricting to cases at the higher end of the spectrum in terms of severity. Thus, the distributions of traditional CV risk factors and potential confounders were similar between lupus and controls, and such comparability in known CV risk factors enabled more focused investigation of novel risk factors. Further, two major CVD risk factors – smoking and diabetes – were exclusion criteria and thus did not complicate the interpretation of our results. Various soluble markers (*e.g.*, adhesion molecules, hsCRP) reported as associated with vascular damage in other conditions did not correlate with atherosclerosis or endothelial dysfunction in our population.

Differences between our findings and other studies [45], [46] may be related to patient population variations, including race/ethnicity, age, and disease activity, or to the CV surrogate outcomes evaluated. With the majority of our population being female (reflecting the underlying preponderance of SLE among women), our findings are not directly comparable to those from studies involving a larger proportion of males. Likewise, our population was relatively homogeneous in terms of race/ethnicity, potentially limiting generalizability of our results to non-white populations. SLE patients in this study, with overall low disease activity and low Framingham scores, had significantly impaired FMD versus controls, and non-significantly increased CIMT and coronary calcification. The lack of significant differences between cases and controls for CIMT and coronary calcification may reflect the relatively young age of the study population, which was by design in order to avert the confounding effects of the postmenopausal state and comorbidities on endothelial function. Low disease activity may have also contributed to these findings.

Longitudinal research will be necessary to characterize patterns of IFIG induction over time (*e.g.*, chronic versus acute/transient activation) in both SLE and non-SLE populations, and to elucidate factors underlying the detected interaction between IFIGs and case-control status. Further, the impact of treatments, including immunosuppressives and statins, on IFIG induction needs to be studied in detail. In an observational study such as ours, it is difficult to directly assess the impact of medications, and confounding by indication is a concern. Therefore, it would be interesting to evaluate type I IFN signatures in ancillary studies to randomized controlled trials. It will also be important to examine the role of type I IFNs in cohorts of patients with more severe lupus manifestations, as the patient population we studied was well-controlled. Future studies need to distinguish the roles of type I IFN pathway components, and at various stages in the natural history of CVD.

In conclusion, type I IFNs are independently associated with both functional and anatomic markers of subclinical CVD. Our results support the hypothesis that type I IFNs promote accelerated atherosclerosis in SLE, and that excess CVD risk in SLE patients is in part due to factors that operate uniquely in SLE patients as opposed to controls. Future studies should address if blockade of type I IFN pathways early in the natural history of disease leads to a reduction of CVD in SLE and, potentially, in other autoimmune diseases.
